# Circulating miRNA profiles and the risk of hemorrhagic transformation after thrombolytic treatment of acute ischemic stroke: a pilot study

**DOI:** 10.3389/fneur.2024.1399345

**Published:** 2024-06-12

**Authors:** Marcin Stańczak, Adam Wyszomirski, Paulina Słonimska, Barbara Kołodziej, Bartosz Jabłoński, Anna Stanisławska-Sachadyn, Bartosz Karaszewski

**Affiliations:** ^1^Department of Adult Neurology, Faculty of Medicine, Medical University of Gdańsk, Gdańsk, Poland; ^2^Department of Adult Neurology, University Clinical Center, Gdańsk, Poland; ^3^Brain Diseases Centre, Medical University of Gdańsk, Gdańsk, Poland; ^4^Laboratory for Regenerative Biotechnology, Department of Biotechnology and Microbiology, Gdańsk University of Technology, Gdańsk, Poland; ^5^Department of Biotechnology and Microbiology, Gdańsk University of Technology, Gdańsk, Poland; ^6^BioTechMed Center, Gdańsk University of Technology, Gdańsk, Poland

**Keywords:** microRNA, miR, ischemic stroke, hemorrhagic transformation, thrombolysis, biomarker

## Abstract

**Background:**

Hemorrhagic transformation (HT) in acute ischemic stroke is likely to occur in patients treated with intravenous thrombolysis (IVT) and may lead to neurological deterioration and symptomatic intracranial hemorrhage (sICH). Despite the complex inclusion and exclusion criteria for IVT and some useful tools to stratify HT risk, sICH still occurs in approximately 6% of patients because some of the risk factors for this complication remain unknown.

**Objective:**

This study aimed to explore whether there are any differences in circulating microRNA (miRNA) profiles between patients who develop HT after thrombolysis and those who do not.

**Methods:**

Using qPCR, we quantified the expression of 84 miRNAs in plasma samples collected prior to thrombolytic treatment from 10 individuals who eventually developed HT and 10 patients who did not. For miRNAs that were downregulated (fold change (FC) <0.67) or upregulated (FC >1.5) with *p* < 0.10, we investigated the tissue specificity and performed KEGG pathway annotation using bioinformatics tools. Owing to the small patient sample size, instead of multivariate analysis with all major known HT risk factors, we matched the results with the admission NIHSS scores only.

**Results:**

We observed trends towards downregulation of miR-1-3p, miR-133a-3p, miR-133b and miR-376c-3p, and upregulation of miR-7-5p, miR-17-3p, and miR-296-5p. Previously, the upregulated miR-7-5p was found to be highly expressed in the brain, whereas miR-1, miR-133a-3p and miR-133b appeared to be specific to the muscles and myocardium.

**Conclusion:**

miRNA profiles tend to differ between patients who develop HT and those who do not, suggesting that miRNA profiling, likely in association with other omics approaches, may increase the current power of tools predicting thrombolysis-associated sICH in acute ischemic stroke patients. This study represents a free hypothesis-approach pilot study as a continuation from our previous work. Herein, we showed that applying mathematical analyses to extract information from raw big data may result in the identification of new pathophysiological pathways and may complete standard design works.

## 1 Introduction

Ischemic stroke was found to have an incidence of 7.6 million individuals worldwide in 2019, resulting in 63.48 million disability-adjusted life years (DALYs) and 3.29 million deaths. Ischemic stroke is a devastating neurological condition characterized by brain tissue damage caused by sudden obstruction of blood flow in the cerebral arteries (1, 2). Treatment in the acute phase aims to restore blood flow through intravenous thrombolysis and mechanical thrombectomy. The former method, which is used in up to 25% of patients, involves the administration of tissue-type plasminogen activator (rtPA), which promotes the formation of plasmin, a proteolytic enzyme. Plasmin breaks the crosslinks between fibrin molecules, leading to thrombus dissolution and restoration of blood flow (3, 4).

Hemorrhagic transformation (HT), which involves the extravasation of blood across a disrupted blood–brain barrier into the brain parenchyma, is one of the most common complications of ischemic stroke (5). According to the European Cooperative Acute Stroke Study (ECASS), HT can be categorized based on its intensity and radiological features into small petechial hemorrhagic infarction (HI1), confluent petechial hemorrhagic infarction (HI2), small parenchymal hemorrhage (PH1) (<30% infarct, mild mass effect), and large parenchymal hemorrhage (PH2, >30% infarct, marked mass effect) (6). Depending on its severity, HT may remain asymptomatic; however, if it is sufficiently large to exert a mass effect on brain tissue outside the infarct, it may cause neurological deterioration (7). Autopsy studies revealed hemorrhagic transformations in 18–42% of patients with acute ischemic stroke, and clinical assessment indicated symptomatic intracerebral hemorrhage after intravenous thrombolysis in approximately 6% of patients (8, 9).

Several studies aim at pinpointing reliable predictors of hemorrhagic transformation. The established clinical risk factors include baseline National Institutes of Health Stroke Scale (NIHSS) score, systolic and diastolic blood pressure, atrial fibrillation, antiplatelets use, age, and time from onset to treatment and hyperglycemia among others (10, 11). Radiological determinants of increased risk of hemorrhagic transformation include a large infarct size, early ischemic changes visible on computed tomography (CT), and absent or poor collaterals (10, 12). Among identified blood biomarkers, matrix metalloproteinase-9 (MMP-9), ferritin, and cellular fibronectin (c-Fn), as well as the neutrophil-to-lymphocyte ratio (NLR) and high-density lipoprotein (HDL), have been extensively studied across multiple experiments (13, 14).

Recent advances in artificial intelligence (AI) and omics have fostered their application in the search for novel HT biomarkers and predictive models. Machine learning methods have been used to develop predictive models based on clinical data and laboratory test results (15). In our previous study, we explored a hypothesis-free approach using MS proteomic data to identify new biomarkers (16). In that study, 15 proteins detected in the blood collected prior to rtPA treatment were unique to patients who developed HT.

MicroRNAs (miRNAs) are small non-coding RNA molecules composed of approximately 22 nucleotides that are known for their regulatory roles in various biological processes, mainly through the post-transcriptional regulation of gene expression (17). Their stability and detectability in various tissues, including blood, have attracted significant attention in the last decade, leading to their exploration as potential diagnostic and prognostic biomarkers, particularly in oncology (18). Circulating miRNAs have also emerged as valuable tools in stroke medicine. Numerous studies have identified miRNAs as diagnostic markers for ischemic stroke, with hsa-let-7e-5p, hsa-miR-124-3p, hsa-miR-17-5p, and hsa-miR-185-5p showing consistent differential expression (19). Furthermore, the combination of miR-124-3p, miR-125b-5p, and miR-192-5p expression has been shown to predict the extent of neurological deterioration in ischemic stroke patients treated with rtPA (20). In another study, miR-21-5p, miR-206, and miR-3123 were implicated in predicting the risk of hemorrhagic transformation in patients with cardioembolic stroke (21). Additionally, the assessment of RNA markers, including miRNA-23a, miRNA-193a, miRNA-128, miRNA-99a, miRNA-let-7a, miRNA-494, miRNA-424, and the long non-coding (lnc)RNA H19, has been shown to improve the prediction of symptomatic intracranial hemorrhage (sICH) after rtPA (22).

The findings of the above studies suggest that quantitative miRNA and proteomic data may increase the current power of the tools for predicting thrombolysis-associated sICH in patients with acute ischemic stroke [as we showed in our previous study (16)]. However, the main objective of the presented studies is to demonstrate a methodology for and the feasibility of such an approach. This pilot study only aimed to identify potential miRNAs indicative of an increased risk of HT occurrence.

## 2 Methods

### 2.1 Study population

This research is a continuation of our previous study on biomarkers of rtPA-associated intracranial hemorrhage in acute ischemic stroke (16). Participants were recruited between March 2019 and April 2022 from among patients at the Stroke Comprehensive Center of the Department of Adult Neurology, University Clinical Center, Medical University of Gdansk.

Before recruitment, approval was obtained from The Independent Bioethics Committee for Scientific Research at the Medical University of Gdańsk in Poland. This study was conducted in accordance with the principles outlined in the World Medical Association Declaration of Helsinki. All subjects were informed of the purpose and course of the study and signed an informed consent form.

For patients with acute stroke, a pretreatment MRI protocol (described in Section 2.2) was performed, in addition to the collection of two blood samples: plasma in an EDTA-coated tube and serum in a silica clot activator (Becton Dickinson Vacutainer). However, only plasma samples were used for further analyses. Upon confirmation of ischemic stroke diagnosis and patient adherence to thrombolysis inclusion criteria according to the stroke guidelines, alteplase was administered intravenously at a standard dose of 0.9 mg/kg. Subsequently, patients were assigned to either the HT or control group, based on follow-up MRI scans on day 5–9 after the treatment. In the initial cohort of 94 patients admitted for acute stroke, 56 received intravenous rtPA, met the standard inclusion and exclusion criteria, and had no contraindications for MRI. Due to limited funding during this phase of the study, 10 patients with HT were selected for further analysis and matched with 10 non-HT controls based on comparable risk factors for hemorrhagic transformation (NIHSS score, Oxfordshire Community Stroke Project classification) and similar miRNA profiles (age and sex).

The Propensity Score Matching method, which incorporates the k-nearest neighbor (k-NN) algorithm without replacement, was used to select a well-matched control group (non-HT) with a distribution of baseline characteristics similar to that of the HT group. The analysis was conducted using the MatchIt library in R software (23).

### 2.2 Brain imaging

Pretreatment MRI was performed using a Siemens Healthcare GOBrain application on a 1.5 T MRI-scanner (Siemens Magnetom Aera), comprising axial T2-weighted fluid attenuation inversion recovery, axial diffusion-weighted imaging (*b*-values 0,800 s/mm) with apparent diffusion coefficient maps, axial T2*-weighted, and sagittal T1-weighted images. These scans were used to assess the infarct volume and were analyzed by radiologists using the syngo.via software. The follow-up MRI scan was taken using the same 1.5 T Siemens device, but the protocol was expanded compared to the initial one and additionally included the following sequences: axial susceptibility-weighted imaging, sagittal T2-weighted, axial diffusion tensor imaging, and 3D axial T1-weighted sequences.

### 2.3 miRNA quantification

Plasma samples previously stored at deep-freezing temperatures (−80°C) were used for miRNA quantification. Briefly, plasma miRNAs were isolated using the miRNeasy Serum/Plasma Advanced Kit (Cat. no. 217204; Qiagen, Hilden, Germany). cDNA was synthesized using a miRCURY LNA RT Kit (Cat. no. 339340; Qiagen, Hilden, Germany). Each sample contained miRNA corresponding to 16 μL of plasma sample, 1 x buffer, 1 x miRCURY RT Enzyme mix, synthetic RNA spike-ins. All cDNA samples were synthesized from a master mix of reagents. Samples were incubated for 60 min at 42°C followed by incubation for 5 min at 95°C. The efficiency of miRNA isolation and cDNA synthesis was evaluated by PCR analysis of exogenous synthetic miRNAs (Cat. no. 339390; Qiagen, Hilden, Germany). DNA isolation and cDNA synthesis were successful in all samples.

The quantification of 84 miRNAs was performed using the Serum/Plasma Small Focus miRCURY LNA Panel (cat. no. 339325; Qiagen), which comprised 84 human miRNAs curated by the manufacturer based on their highest expression in serum and plasma samples obtained from healthy individuals and those afflicted with various conditions. The LC96 Roche platform was used. The same panel was used to determine the occurrence of hemolysis. The determinant that indicated an increased risk of hemolysis exceeded the value of dCq = 7, which was the difference between the value of miR-23a, which is constant in the blood, and the value of miR-451a, which is specific to red blood cells. The miRNA analyses were performed at the Department of Molecular Biotechnology and Microbiology of Gdansk University of Technology.

### 2.4 Statistical analysis and results exploration

Normalized miRNA expression was calculated as 2^-(Cq(miRNA) - Cq(mean of references))^. The fold change was calculated for each miRNA as the quotient of the mean normalized expression in the HT and non-HT groups.

The Yuen–Welch t-test permutation was applied to compare the trimmed means of the two study groups. The number of permutations was set to 1,000 with a random seed of 123 in the R program. A 10%-trimmed mean value was established. To address the issue of multiple hypothesis testing, *p*-values were adjusted using the Benjamini-Hochberg correction method. All statistical analyses were performed using R software version 3.6.3.

A multifactor analysis allowed us to control for confounding factors. However, the sample size in the present study was too small to achieve statistical validity; (24) therefore, we limited our single-factor analysis.

To assess miRNA tissue/organ specificity, we utilized the TissueAtlas (24). Subsequently, the significant miRNAs were subjected to functional analysis using DIANA mirPath v.3 (25). To identify miRNA target genes, we selected TarBase v.7.0, a database of experimentally validated miRNA-gene interactions (26). The tool was set to annotate target genes to Kyoto Encyclopedia of Genes and Genomes (KEGG) pathways, with results merging set to a pathway union (27).

## 3 Results

### 3.1 Patients’ characteristic

Of 75 patients with acute ischemic stroke treated with intravenous thrombolysis, 25 were excluded because of incomplete data. Among the remaining 50 patients, HT was detected in 10. All patients with HT experienced stroke in the anterior circulation territory and none had lacunar strokes. As this was only the pilot phase of the study, HT was not differentiated into symptomatic or asymptomatic. Ten patients with anterior circulation and non-lacunar stroke who developed HT were matched with 10 patients without HT, as described in the Methods section. The clinical and demographic characteristics of the patients selected for further investigation are presented in Table 1. The complete dataset is shown in Supplementary Table S1.

**Table 1:** Demographical and clinical characteristics of patients selected for miRNA analysis.

	Patients with HT (*n* = 10)	Patients without HT (*n* = 10)
Age, median (IQR)	77.5 (60.75–86.25)	80 (76.00–85.5)
Sex, female /male, *n* (%)	3 (30%) / 7 (70%)	5 (50%) / 5 (50%)
NIHSS on admission, median (IQR)	8 (4.25–11.00)	5 (4.25–10.75)
Stroke subtype according to OCSP, n (%)		
PACS	7 (70%)	7 (70%)
TACS	3 (30%)	3 (30%)
Baseline infarct volume (IQR)	12.14 (0.0–19.35)	25.92 (11.30–42,52)
Systolic blood pressure on admission, median (IQR)	159.5 (150.00–165.25)	167 (150.5–179.50)
Diastolic blood pressure on admission, median (IQR)	79 (71.25–90.00)	82.5 (74.5–90.00)
Comorbidities, *n* (%)		
Atrial Fibrillation	1 (10%)	4 (40%)
Diabetes	2 (20%)	1 (10%)
Hypertension	9 (90%)	8 (80%)
Hyperlipidemia	9 (90%)	4 (40%)
Antiplatelets use	4 (40%)	6 (60%)
Active smoking	3 (30%)	4 (40%)
Type of HT according to ECASS, *n* (%)		
HI1	7 (70%)	–
HI2	2 (20%)	–
PH1	1 (10%)	–
PH2	0 (0%)	–
NIHSS on discharge, median (IQR)	1.5 (0–2.75)	0.5 (0–1.75)

IQR, interquartile range; OCSP, oxfordshire community stroke project (classification); PACS, partial anterior circulation stroke; TACS, total anterior circulation stroke; ECASS, European cooperative acute stroke study (classification); HI1, hemorrhagic infarction type 1; HI2, hemorrhagic infarction type 2; PH1, parenchymal hematoma type 1.

### 3.2 miRNA isolation and cDNA synthesis

To estimate the RNA isolation efficiency, UniSp2, UniSp4, and UniSp5 spike-ins were added to the plasma samples, which allowed for the comparison of RNA isolation between samples after RT-qPCR. RNA isolation efficiency remained stable across all samples, with UniSp2 presenting a minimum quantification cycle (Cq) value of 22.01 and a maximum Cq value of 19.47 for samples 108 and 160, respectively. In addition, UniSp4 (present at a 100-fold lower concentration than UniSp2) and UniSp5 (present at a 100-fold lower concentration than UniSp4) were detected in all samples. Similarly, cDNA synthesis efficiency was assessed using the UniSp6 spike-in, which was amplified with the min Cq value of 19.11 and max Cq value of 18.56 among all samples.

### 3.3 Hemolysis in samples

**Figure 1:**
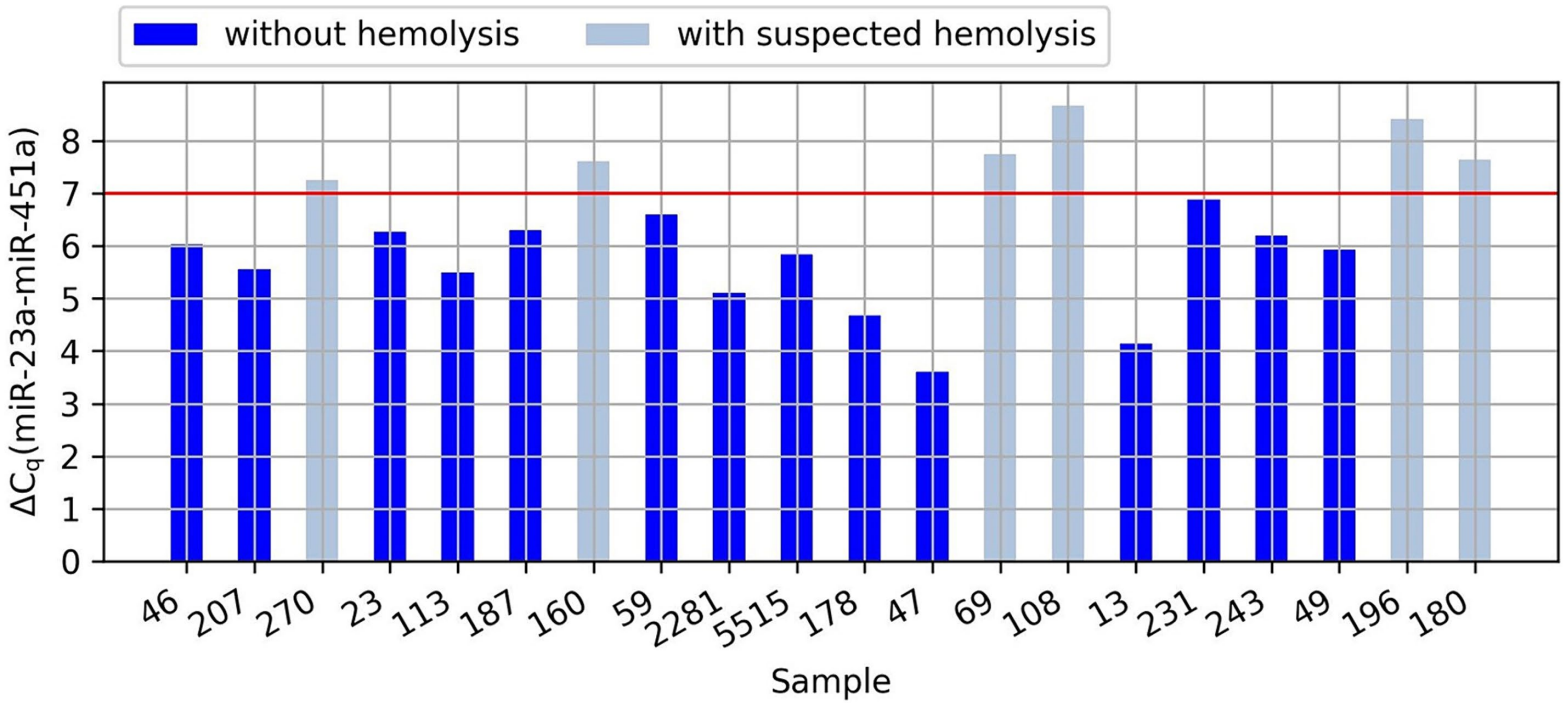
Graph showing the difference in the Cq values of miR-23a and miR-451a (dCq) in the analyzed samples. The red line indicates the value of dCq above which there is an increased risk of hemolysis.

Based on the analysis of the differences in the Cq values of miR-23a and miR-451a, it was noticed that in six samples, the probability of hemolysis increased (the dCq value was higher than 7). In four samples we observed the hemolysis marker at values between 7.26 and 7.76 and in two samples at values of 8.43 and 8.68 (the exact values of dCq for each sample are presented in the Figure 1). However, since low levels of hemolysis are difficult or impossible to eliminate in clinical practice and we cannot determine the degree of hemolysis based on these results, while our samples were not highly hemolyzed, we decided to continue the research using these samples.

### 3.4 miRNA differential expression

The miRNAs hsa-miR-30c-5p, hsa-miR-103a-3p, and hsa-miR-23a-3p were selected as reference miRNAs to calculate the relative expression of plasma miRNAs. Normalized results of miRNA quantification are provided in Supplementary Table S2. When the mean Cq of the reference miRNAs was calculated, sample no. 47 was found to be an outlier. The mean Cq value for the reference miRNAs was 34.67, while the lowest mean Cq value among the other samples was 30.03. Thus, the levels of miRNAs in this sample were notably lower, which resulted in the lack of detection of several plasma miRNAs. This led us to exclude sample 47 from further analyses.

**Figure 2:**
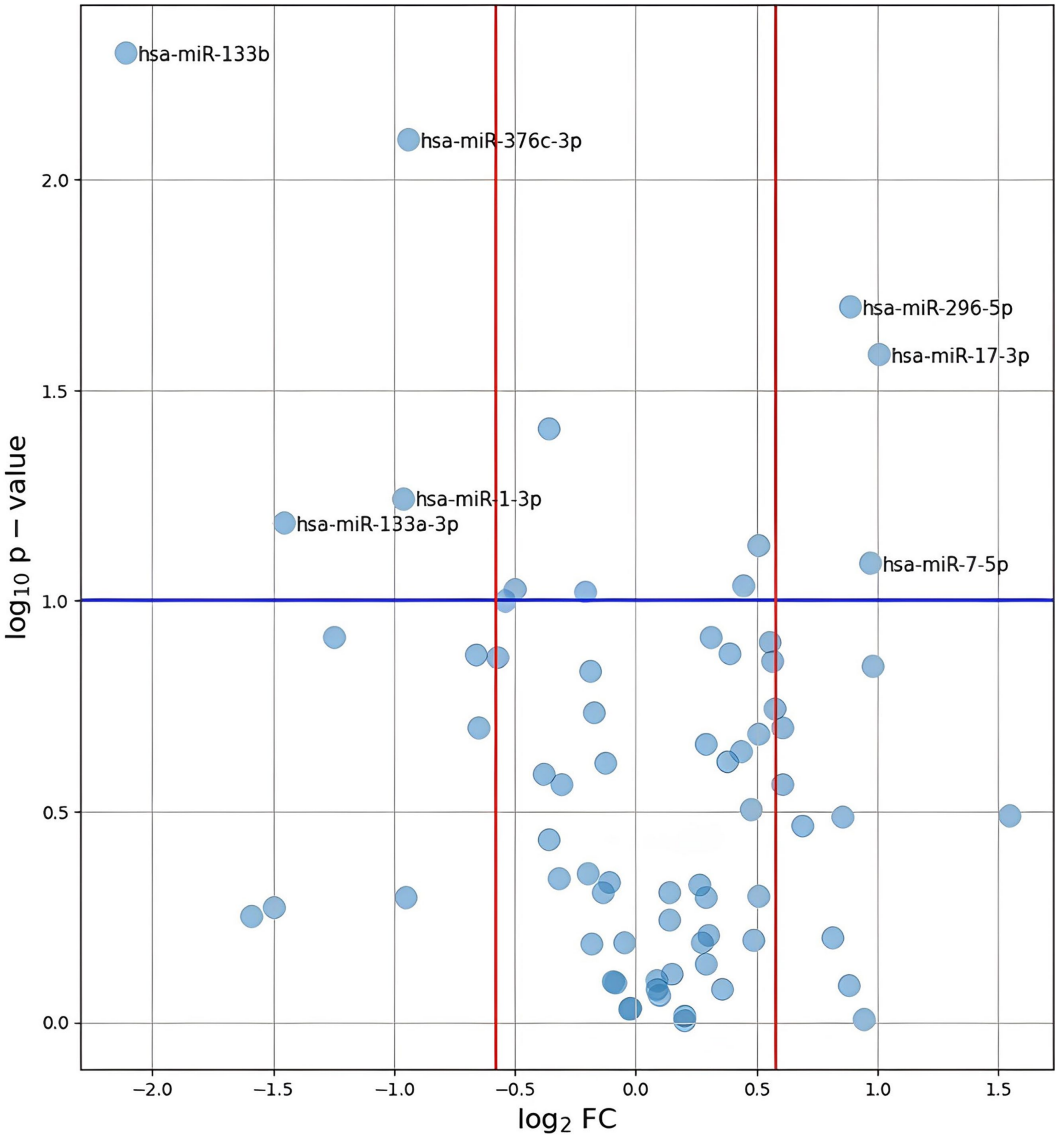
Plot of log2(FC) against –log10(*p*-value) for all analyzed miRNAs. The values presented on the plot were calculated using the *p*-values obtained after performing Permutation Yuen-Welch *t* test. A blue line represents the level of *p*-value = 0.1. Red lines indicate the range of FC < 0, 67 or > 1, 5.

Of the 84 assessed miRNAs, 12 were excluded from further analyses due to missing data in more than 50% of the samples and one was excluded due to non-specific amplification. In our study, “missing data,” referred to situations in which the concentration of genetic material was so low that we were unable to detect it. Following normalization of miRNA levels, differential expression was calculated. The results are shown in Figure 2. Thirteen miRNAs reached a Permutation Yuen-Welch *t*-test *p*-value <0.10, with five having a p-value <0.05. However, after Benjamini and Hochberg correction for multiple testing problems, none of the analyzed miRNAs had a p-value <0.05.

Regarding the magnitude of difference between the HT and non-HT groups, miRNAs with a fold change (FC) in relative expression (FC) <0.67 were considered downregulated in HT, while miRNAs with FC >1.5 were deemed upregulated. Owing to the nature of the pilot study, the level of significance was set to 0.10. Using the commonly encountered value of 0.05 could potentially result in the pilot study being underpowered (28). The results of the statistical analysis of all miRNAs with a complete set of data are provided in Supplementary Table S3. Sources for the analysis are avaliable in online repository: https://github.com/barbara-kolodziej-gumed/Circulating-miRNA-profiles-and-the-risk-of-HT-after-rtPA.

The analysis included two previously described stroke biomarkers, hsa-miR-124-3p and hsa-miR-17-5p. The concentration of hsa-miR-124-3p was so low in 12 samples that quantitative determination was not possible. For hsa-miR-17-5p, no statistically significant differences were found between the HT and non-HT groups.

**Figure 3:**
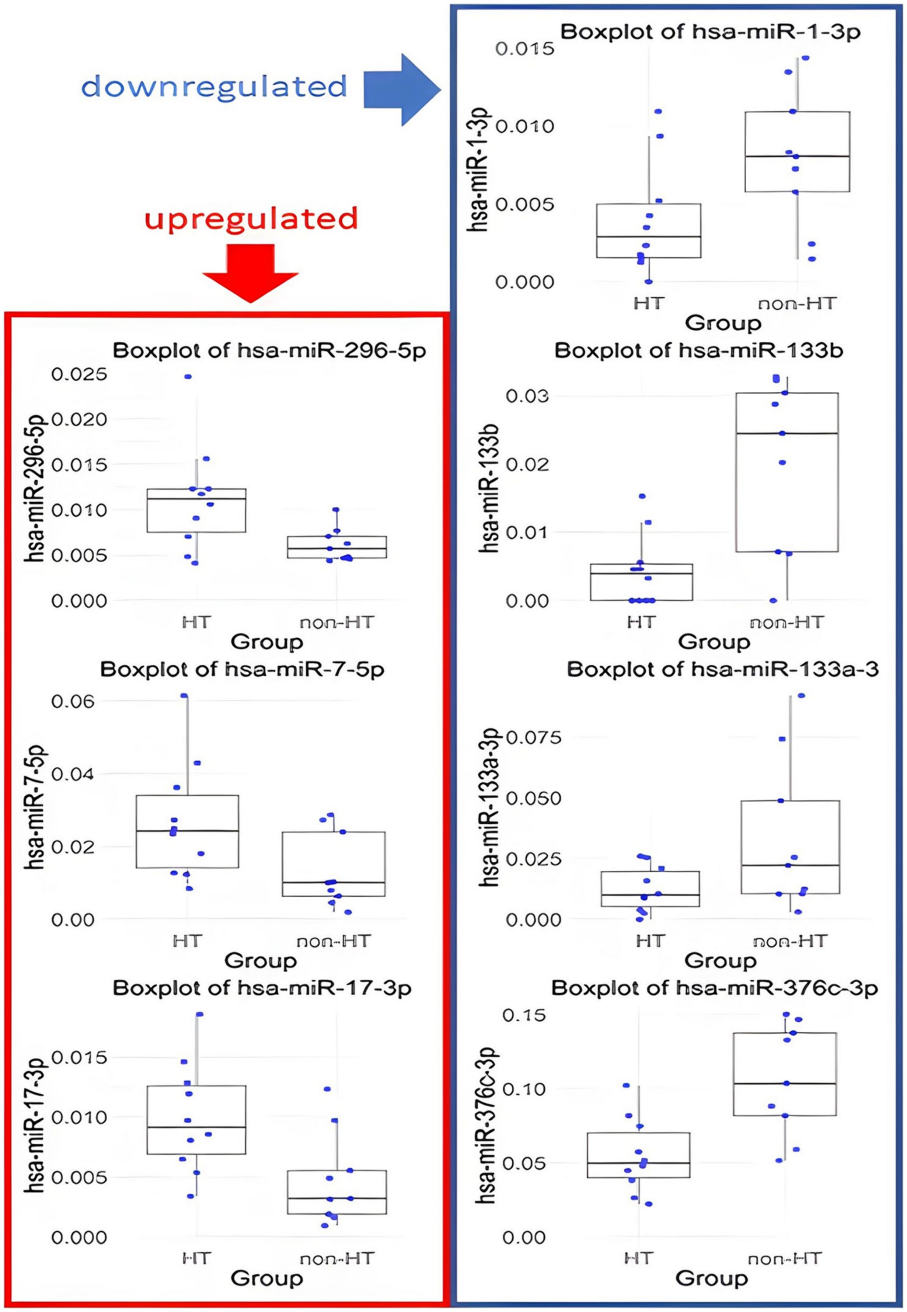
Box plots of miRNA expression in HT and non-HT group. Only results with *p* < 0.1 and FC >1.5 or < 0.67 are presented.

MiRNAs with *p*-values <0.1 and FC <0.67 or > 1.5 are listed in Table 2. Specifically, hsa-miR-133b, hsa-miR-376c-3p, hsa-miR-133a-3p, and hsa-miR-1-3p were downregulated, whereas hsa-miR-17-3p, hsa-miR-296-5p and hsa-miR-7-5p were upregulated in the ICH group. The distributions of their values in the HT and non-HT groups are shown in Figure 3.

**Table 2:** miRNAs with *p* < 0.05 and FC >1.5 or < 0.67.

miRNA	FC in relative expression of miRNAs in HT compared to non-HT	Permutation Yuen-Welch *t* test *p*-value	Adjusted *p*-value
hsa-miR-133b	0.23	0.005	0.284
hsa-miR-376c-3p	0.52	0.008	0.284
hsa-miR-296-5p	1.85	0.020	0.462
hsa-miR-17-3p	2.01	0.026	0.462
hsa-miR- 1-3p	0.51	0.057	0.474
hsa-miR-133a-3p	0.36	0.065	0.474
hsa-miR-7-5p	1.96	0.081	0.474

Downregulated miRNAs are marked in blue, upregulated ones in red.

Next, the TissueAtlas was used to determine whether these seven miRNAs exhibited specificity to any organ or tissue. Specifically, miR-133b, miR-1-3p, and miR133a-5p appeared to be predominantly present in muscles and myocardium, miR-7-5p was found to be abundant in the brain, miR-376c-3p in the brain, bone, and tunica albuginea, and miR-17-3p in muscle and thyroid tissues, whereas miR-296-5p levels were uniform across all the investigated tissues (Supplementary Table S4).

**Figure 4:**
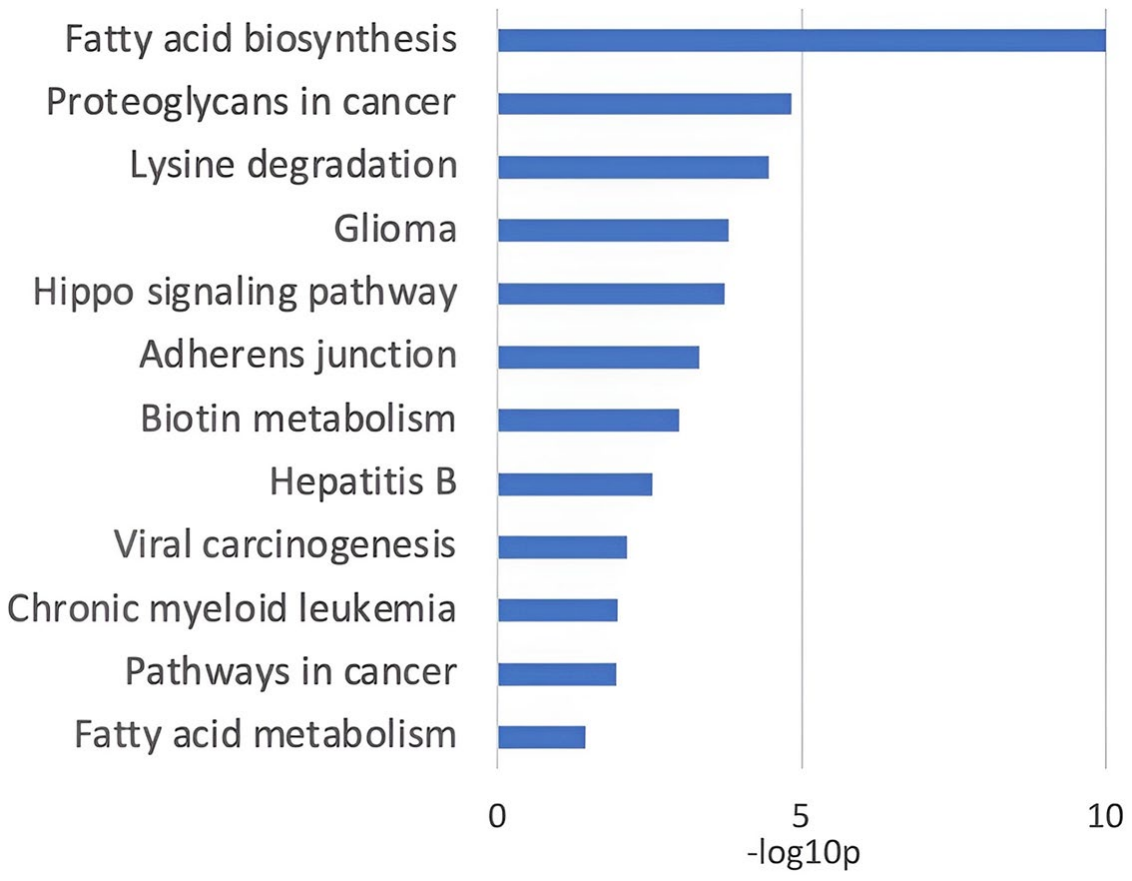
KEGG pathways annotated to the 7 investigated miRNAs.

Subsequently, the DIANA miR path tool was used to assess the potential functional roles of the seven miRNAs. The KEGG pathways significantly associated with the investigated miRNAs are shown in Figure 4. These pathways include those associated with cell adhesion molecules, fatty acid synthesis, and metabolism, as well as those associated with signal transduction, amino acid metabolism, and pathways explored in carcinogenesis.

## 4 Discussion

This pilot study highlighted the differences in circulating miRNA profiles between patients who developed HT and those who did not. We hypothesized that miRNA profiling and other omic approaches may increase the predictive power of thrombolysis-associated sICH in acute ischemic stroke; however, this requires further investigation. This study was a free hypothesis-approach pilot study based on previous work from our group, in which we showed that applying mathematical analyses to extract information from raw big data may result in the identification of new pathophysiological pathways and may complete standard design work.

Despite the low number of patients investigated in this study and the restricted miRNA panel, to the best of our knowledge, this study still reveals the broadest panel of miRNAs that might play a role in hemorrhagic transformation associated with the thrombolytic treatment of acute ischemic stroke.

The miRNAs miRNA-1, −133a and −133b, which were downregulated in HT patients, are known to be present in abundance in both skeletal and cardiac muscle (29). They have further been reported to be elevated in the circulation following a myocardial infarction (30). Notably, miR-133b alone has been found to be a risk factor for cardiovascular disease (31). Another miRNA downregulated in HT, miR-376c, has been extensively studied as a cancer modulator, with evidence supporting a role in both cancer suppression and progression (32). In neonatal hypoxic–ischemic encephalopathy, miR-376c demonstrates protective effects against oxygen–glucose deprivation-induced cell injury (33). One of the upregulated miRNAs, miR-296-5p, has previously been found to be present at high levels in deep venous thrombosis patients. Interestingly, in a mouse model, its elevation inhibited deep venous thrombosis by suppressing S100A4 expression, which influences the release of prothrombosis-related factors (34). Therefore, the observed increase in the level of this miRNA in patients with HT after intravenous thrombolysis in our study is of particular interest. MiR-17-3p is a member of the miR-17/92 cluster and plays a role in cell cycle regulation, proliferation, and apoptosis. It is frequently dysregulated in cardiovascular, immune, and neurodegenerative diseases (35). Notably, in a mouse model, miR-17-3p overexpression suppressed cardiomyocyte apoptosis induced by oxygen–glucose deprivation and reperfusion (36).

The last upregulated miRNA, miR-7-5p, is highly expressed in the brain and is involved in cerebral cortex development. It has also been shown to play a role in neurodegenerative diseases, neuroinflammation, and mental disorders (37). In rodent models, miR-7-5p expression was found to be downregulated in the brain following focal ischemia. Interestingly, administration of miR-7 mimic resulted in smaller infarct volume and better functional recovery, supposedly by repressing α-synuclein (38). Among the imRNAs excluded from the differential expression analysis due to low signal intensity, hsa-miR-124-3p was identified as potentially important. This miRNA is downregulated in ischemic stroke (39), which could explain its undetectable expression in our samples.

The detected trends in miRNA expression aligned with patterns observed in various cardiovascular diseases and the pathophysiological mechanisms associated with ischemic stroke. Implementing our proposed methodology in a large-scale study could facilitate the identification of significant biomarkers indicative of the risk of hemorrhagic transformation. Broadening the spectrum of analyzed miRNAs through either an expanded qPCR panel or small RNA sequencing would further enrich the study and potentially deepen our understanding of the pathophysiology of hemorrhagic transformation.

### 4.1 Limitations

In this miRNA analysis, owing to the small sample size, a multiple regression model that included the baseline characteristics of the patients used in the matching process was not calculated. The small size of the recruited study group resulted in none of the adjusted *p*-values being <0.1. Therefore, based on the obtained results, it is not possible to definitively determine which miRNAs may serve as markers of HT after rtPA treatment for stroke. This article is a pilot study and primarily serves as a description of miRNA measurement methods in clinical practice. It should also be noted that the quality of the results may also be influenced by hemolysis, which was observed in six samples. However, in clinical practice, the risks of suboptimal collection and handling of biological materials must be considered. Therefore, these samples were included in our analysis.

## 5 Conclusion

The quantitative determination of miRNA expression in HT and non-HT patients serves as a complement to our previous analysis (16) and may potentially expand the predictive power of currently used calculators for thrombolysis-associated HT in acute ischemic stroke based on clinical and neuroimaging data; however, further studies are needed to verify this idea. We found differences in the blood concentrations of seven miRNAs, and further analysis of the available miRNA databases revealed that miR-296-5p was most directly linked to the occurrence of HT. However, owing to the small sample size, these results may only be considered as a pilot for any major study. Overall, this study shows that analyzing datasets for a sufficiently large number of patients using a hypothesis-free approach could be used to identify complex pathophysiological associations that may be unachievable in standard-design studies.

## Data availability statement

The datasets presented in this study can be found in online repositories. The names of the repository/repositories and accession number(s) can be found in the article/Supplementary material.

## Ethics statement

The studies involving humans were approved by The Independent Bioethics Committee for Scientific Research at the Medical University of Gdańsk, Poland. The studies were conducted in accordance with the local legislation and institutional requirements. The participants provided their written informed consent to participate in this study.

## Author contributions

MS: Formal analysis, Investigation, Project administration, Resources, Visualization, Writing – original draft. AW: Formal analysis, Writing – original draft. PS: Investigation, Writing – review & editing. BKo: Project administration, Visualization, Writing – original draft. AS-S: Writing – original draft, Formal analysis, Investigation, Methodology. BKa: Conceptualization, Funding acquisition, Methodology, Supervision, Writing – review & editing. BJ: Writing – review & editing, Data curation.

## Supplementary material

SUPPLEMENTARY TABLE S1 Clinical and neuroimaging data of 20 stroke patients with and without hemorrhagic transformation.

SUPPLEMENTARY TABLE S2 Normalized results of miRNA quantification.

SUPPLEMENTARY TABLE S3 MicroRNA comparison between study groups.

SUPPLEMENTARY TABLE S4 Expression of selected miRNAs in various organs according to the TissueAtlas.
