# Supplementary material for: Circulating miRNA profiles and the risk of hemorrhagic transformation after thrombolytic treatment of acute ischemic stroke: a pilot study
Source: Front Neurol. 2024 Jun 12;15:1399345. doi: 10.3389/fneur.2024.1399345 (PMC11210454; doi:10.3389/fneur.2024.1399345)
Supplement: SUPPLEMENTARY TABLE S3 — MicroRNA comparison between study groups. [file Table_3.DOCX]

Supplementary Table 3 MicroRNA comparison between study groups**

| **Variable** | **Ich**, N = 10 | **Non Ich**, N = 9 | **Ratio of means** | **P-value**^1^ | **Multiplicity adjusted p-value**^2^ |
| --- | --- | --- | --- | --- | --- |
| **hsa-let-7a-5p** |  |  | 0.887 | 0.184 | 0.542 |
| N | 10 | 9 |  |  |  |
| N missing | 0 | 0 |  |  |  |
| Mean | 0.86 | 0.97 |  |  |  |
| Median (Q1-Q3) | 0.88 (0.79-0.93) | 0.90 (0.87-0.98) |  |  |  |
| Min - Max | 0.66 - 1.01 | 0.81 - 1.35 |  |  |  |
| **hsa-miR-1-3p** |  |  | 0.512 | 0.057 | 0.474 |
| N | 10 | 9 |  |  |  |
| N missing | 0 | 0 |  |  |  |
| Mean | 0.004 | 0.008 |  |  |  |
| Median (Q1-Q3) | 0.003 (0.002-0.005) | 0.008 (0.006-0.011) |  |  |  |
| Min - Max | 0.000 - 0.011 | 0.001 - 0.014 |  |  |  |
| **hsa-miR-100-5p** |  |  | 0.936 | 0.801 | 0.912 |
| N | 9 | 9 |  |  |  |
| N missing | 1 | 0 |  |  |  |
| Mean | 0.020 | 0.022 |  |  |  |
| Median (Q1-Q3) | 0.012 (0.006-0.027) | 0.018 (0.014-0.033) |  |  |  |
| Min - Max | 0.004 - 0.055 | 0.003 - 0.045 |  |  |  |
| **hsa-miR-106b-5p** |  |  | 1.35 | 0.227 | 0.554 |
| N | 10 | 9 |  |  |  |
| N missing | 0 | 0 |  |  |  |
| Mean | 1.64 | 1.21 |  |  |  |
| Median (Q1-Q3) | 1.54 (1.08-2.32) | 0.98 (0.83-1.21) |  |  |  |
| Min - Max | 0.47 - 2.83 | 0.39 - 2.42 |  |  |  |
| **hsa-miR-10b-5p** |  |  | 1.11 | 0.770 | 0.912 |
| N | 10 | 9 |  |  |  |
| N missing | 0 | 0 |  |  |  |
| Mean | 0.07 | 0.07 |  |  |  |
| Median (Q1-Q3) | 0.07 (0.04-0.08) | 0.07 (0.04-0.10) |  |  |  |
| Min - Max | 0.01 - 0.23 | 0.02 - 0.12 |  |  |  |
| **hsa-miR-122-5p** |  |  | 1.15 | 0.961 | 0.989 |
| N | 10 | 9 |  |  |  |
| N missing | 0 | 0 |  |  |  |
| Mean | 0.31 | 0.27 |  |  |  |
| Median (Q1-Q3) | 0.21 (0.14-0.43) | 0.30 (0.08-0.41) |  |  |  |
| Min - Max | 0.03 - 0.93 | 0.05 - 0.54 |  |  |  |
| **hsa-miR-125b-5p** |  |  | 0.944 | 0.805 | 0.912 |
| N | 10 | 9 |  |  |  |
| N missing | 0 | 0 |  |  |  |
| Mean | 0.06 | 0.06 |  |  |  |
| Median (Q1-Q3) | 0.06 (0.03-0.08) | 0.04 (0.04-0.07) |  |  |  |
| Min - Max | 0.02 - 0.12 | 0.02 - 0.16 |  |  |  |
| **hsa-miR-126-3p** |  |  | 0.917 | 0.242 | 0.554 |
| N | 10 | 9 |  |  |  |
| N missing | 0 | 0 |  |  |  |
| Mean | 1.83 | 2.00 |  |  |  |
| Median (Q1-Q3) | 1.87 (1.65-1.92) | 1.84 (1.64-2.22) |  |  |  |
| Min - Max | 1.42 - 2.57 | 1.54 - 2.81 |  |  |  |
| **hsa-miR-133a-3p** |  |  | 0.364 | 0.065 | 0.474 |
| N | 10 | 9 |  |  |  |
| N missing | 0 | 0 |  |  |  |
| Mean | 0.012 | 0.033 |  |  |  |
| Median (Q1-Q3) | 0.010 (0.005-0.020) | 0.022 (0.011-0.049) |  |  |  |
| Min - Max | 0.000 - 0.026 | 0.003 - 0.092 |  |  |  |
| **hsa-miR-133b** |  |  | 0.231 | 0.005 | 0.284 |
| N | 10 | 9 |  |  |  |
| N missing | 0 | 0 |  |  |  |
| Mean | 0.004 | 0.020 |  |  |  |
| Median (Q1-Q3) | 0.004 (0.000-0.005) | 0.025 (0.007-0.030) |  |  |  |
| Min - Max | 0.000 - 0.015 | 0.000 - 0.033 |  |  |  |
| **hsa-miR-134-5p** |  |  | 1.97 | 0.143 | 0.474 |
| N | 10 | 9 |  |  |  |
| N missing | 0 | 0 |  |  |  |
| Mean | 0.007 | 0.003 |  |  |  |
| Median (Q1-Q3) | 0.007 (0.003-0.009) | 0.001 (0.000-0.004) |  |  |  |
| Min - Max | 0.000 - 0.015 | 0.000 - 0.015 |  |  |  |
| **hsa-miR-141-3p** |  |  | 0.517 | 0.505 | 0.747 |
| N | 10 | 9 |  |  |  |
| N missing | 0 | 0 |  |  |  |
| Mean | 0.006 | 0.010 |  |  |  |
| Median (Q1-Q3) | 0.005 (0.002-0.009) | 0.005 (0.003-0.009) |  |  |  |
| Min - Max | 0.000 - 0.018 | 0.000 - 0.055 |  |  |  |
| **hsa-miR-143-3p** |  |  | 0.707 | 0.094 | 0.474 |
| N | 10 | 9 |  |  |  |
| N missing | 0 | 0 |  |  |  |
| Mean | 0.13 | 0.19 |  |  |  |
| Median (Q1-Q3) | 0.10 (0.07-0.13) | 0.17 (0.07-0.27) |  |  |  |
| Min - Max | 0.04 - 0.46 | 0.06 - 0.38 |  |  |  |
| **hsa-miR-146a-5p** |  |  | 0.925 | 0.465 | 0.747 |
| N | 10 | 9 |  |  |  |
| N missing | 0 | 0 |  |  |  |
| Mean | 0.35 | 0.38 |  |  |  |
| Median (Q1-Q3) | 0.35 (0.27-0.42) | 0.39 (0.31-0.44) |  |  |  |
| Min - Max | 0.25 - 0.48 | 0.23 - 0.48 |  |  |  |
| **hsa-miR-150-5p** |  |  | 0.967 | 0.646 | 0.812 |
| N | 10 | 9 |  |  |  |
| N missing | 0 | 0 |  |  |  |
| Mean | 0.66 | 0.68 |  |  |  |
| Median (Q1-Q3) | 0.54 (0.33-0.88) | 0.64 (0.48-0.68) |  |  |  |
| Min - Max | 0.23 - 1.66 | 0.20 - 1.66 |  |  |  |
| **hsa-miR-155-5p** |  |  | 0.636 | 0.199 | 0.542 |
| N | 10 | 9 |  |  |  |
| N missing | 0 | 0 |  |  |  |
| Mean | 0.008 | 0.013 |  |  |  |
| Median (Q1-Q3) | 0.007 (0.005-0.010) | 0.011 (0.009-0.012) |  |  |  |
| Min - Max | 0.000 - 0.021 | 0.002 - 0.033 |  |  |  |
| **hsa-miR-17-5p** |  |  | 1.36 | 0.092 | 0.474 |
| N | 10 | 9 |  |  |  |
| N missing | 0 | 0 |  |  |  |
| Mean | 2.19 | 1.61 |  |  |  |
| Median (Q1-Q3) | 2.15 (1.95-2.29) | 1.54 (1.33-1.71) |  |  |  |
| Min - Max | 0.70 - 3.96 | 0.77 - 2.81 |  |  |  |
| **hsa-miR-17-3p** |  |  | 2.01 | 0.026 | 0.462 |
| N | 10 | 9 |  |  |  |
| N missing | 0 | 0 |  |  |  |
| Mean | 0.010 | 0.005 |  |  |  |
| Median (Q1-Q3) | 0.009 (0.007-0.013) | 0.003 (0.002-0.006) |  |  |  |
| Min - Max | 0.003 - 0.019 | 0.001 - 0.012 |  |  |  |
| **hsa-miR-18a-5p** |  |  | 1.30 | 0.240 | 0.554 |
| N | 10 | 9 |  |  |  |
| N missing | 0 | 0 |  |  |  |
| Mean | 0.15 | 0.12 |  |  |  |
| Median (Q1-Q3) | 0.16 (0.12-0.17) | 0.12 (0.11-0.13) |  |  |  |
| Min - Max | 0.05 - 0.31 | 0.07 - 0.15 |  |  |  |
| **hsa-miR-192-5p** |  |  | 1.47 | 0.125 | 0.474 |
| N | 10 | 9 |  |  |  |
| N missing | 0 | 0 |  |  |  |
| Mean | 0.14 | 0.09 |  |  |  |
| Median (Q1-Q3) | 0.13 (0.09-0.16) | 0.08 (0.05-0.11) |  |  |  |
| Min - Max | 0.08 - 0.24 | 0.03 - 0.19 |  |  |  |
| **hsa-miR-195-5p** |  |  | 0.881 | 0.652 | 0.812 |
| N | 10 | 9 |  |  |  |
| N missing | 0 | 0 |  |  |  |
| Mean | 0.010 | 0.011 |  |  |  |
| Median (Q1-Q3) | 0.009 (0.009-0.012) | 0.012 (0.010-0.016) |  |  |  |
| Min - Max | 0.002 - 0.017 | 0.000 - 0.019 |  |  |  |
| **hsa-miR-19a-3p** |  |  | 1.49 | 0.180 | 0.542 |
| N | 10 | 9 |  |  |  |
| N missing | 0 | 0 |  |  |  |
| Mean | 8.9 | 5.9 |  |  |  |
| Median (Q1-Q3) | 7.2 (6.5-12.3) | 5.0 (3.3-6.8) |  |  |  |
| Min - Max | 5.0 - 15.1 | 2.2 - 15.1 |  |  |  |
| **hsa-miR-19b-3p** |  |  | 1.39 | 0.312 | 0.626 |
| N | 10 | 9 |  |  |  |
| N missing | 0 | 0 |  |  |  |
| Mean | 7.5 | 5.3 |  |  |  |
| Median (Q1-Q3) | 6.7 (5.4-9.3) | 3.8 (3.0-6.3) |  |  |  |
| Min - Max | 4.0 - 14.1 | 1.9 - 14.1 |  |  |  |
| **hsa-miR-200a-3p** |  |  | 0.983 | 0.924 | 0.968 |
| N | 10 | 9 |  |  |  |
| N missing | 0 | 0 |  |  |  |
| Mean | 0.0035 | 0.0034 |  |  |  |
| Median (Q1-Q3) | 0.0026 (0.0003-0.0062) | 0.0006 (0.0000-0.0075) |  |  |  |
| Min - Max | 0.0000 - 0.0085 | 0.0000 - 0.0085 |  |  |  |
| **hsa-miR-200c-3p** |  |  | 0.354 | 0.534 | 0.774 |
| N | 10 | 9 |  |  |  |
| N missing | 0 | 0 |  |  |  |
| Mean | 0.004 | 0.010 |  |  |  |
| Median (Q1-Q3) | 0.004 (0.000-0.008) | 0.003 (0.000-0.006) |  |  |  |
| Min - Max | 0.000 - 0.011 | 0.000 - 0.066 |  |  |  |
| **hsa-miR-205-5p** |  |  | 0.332 | 0.560 | 0.795 |
| N | 10 | 9 |  |  |  |
| N missing | 0 | 0 |  |  |  |
| Mean | 0.013 | 0.030 |  |  |  |
| Median (Q1-Q3) | 0.011 (0.004-0.016) | 0.005 (0.004-0.008) |  |  |  |
| Min - Max | 0.002 - 0.041 | 0.000 - 0.209 |  |  |  |
| **hsa-miR-20a-5p** |  |  | 1.42 | 0.074 | 0.474 |
| N | 10 | 9 |  |  |  |
| N missing | 0 | 0 |  |  |  |
| Mean | 4.28 | 3.01 |  |  |  |
| Median (Q1-Q3) | 4.19 (3.11-5.55) | 2.77 (2.18-3.43) |  |  |  |
| Min - Max | 1.28 - 7.05 | 1.15 - 5.57 |  |  |  |
| **hsa-miR-21-5p** |  |  | 1.15 | 0.991 | 0.991 |
| N | 10 | 9 |  |  |  |
| N missing | 0 | 0 |  |  |  |
| Mean | 5.82 | 5.12 |  |  |  |
| Median (Q1-Q3) | 5.39 (3.76-6.21) | 4.66 (4.31-6.53) |  |  |  |
| Min - Max | 3.13 - 14.02 | 3.21 - 7.41 |  |  |  |
| **hsa-miR-210-3p** |  |  | 1.21 | 0.646 | 0.812 |
| N | 10 | 9 |  |  |  |
| N missing | 0 | 0 |  |  |  |
| Mean | 0.036 | 0.030 |  |  |  |
| Median (Q1-Q3) | 0.033 (0.021-0.040) | 0.024 (0.019-0.039) |  |  |  |
| Min - Max | 0.005 - 0.075 | 0.009 - 0.062 |  |  |  |
| **hsa-miR-214-3p** |  |  | 0.767 | 0.257 | 0.568 |
| N | 10 | 8 |  |  |  |
| N missing | 0 | 1 |  |  |  |
| Mean | 0.02 | 0.03 |  |  |  |
| Median (Q1-Q3) | 0.00 (0.00-0.01) | 0.01 (0.01-0.02) |  |  |  |
| Min - Max | 0.00 - 0.16 | 0.00 - 0.16 |  |  |  |
| **hsa-miR-215-5p** |  |  | 1.42 | 0.206 | 0.542 |
| N | 10 | 9 |  |  |  |
| N missing | 0 | 0 |  |  |  |
| Mean | 0.10 | 0.07 |  |  |  |
| Median (Q1-Q3) | 0.08 (0.07-0.13) | 0.08 (0.02-0.09) |  |  |  |
| Min - Max | 0.03 - 0.16 | 0.01 - 0.13 |  |  |  |
| **hsa-miR-221-3p** |  |  | 0.871 | 0.442 | 0.747 |
| N | 10 | 9 |  |  |  |
| N missing | 0 | 0 |  |  |  |
| Mean | 0.40 | 0.46 |  |  |  |
| Median (Q1-Q3) | 0.43 (0.37-0.49) | 0.47 (0.43-0.51) |  |  |  |
| Min - Max | 0.15 - 0.54 | 0.28 - 0.62 |  |  |  |
| **hsa-miR-222-3p** |  |  | 1.22 | 0.725 | 0.888 |
| N | 10 | 9 |  |  |  |
| N missing | 0 | 0 |  |  |  |
| Mean | 0.27 | 0.22 |  |  |  |
| Median (Q1-Q3) | 0.22 (0.19-0.29) | 0.22 (0.17-0.30) |  |  |  |
| Min - Max | 0.18 - 0.64 | 0.11 - 0.34 |  |  |  |
| **hsa-miR-223-3p** |  |  | 0.671 | 0.136 | 0.474 |
| N | 10 | 9 |  |  |  |
| N missing | 0 | 0 |  |  |  |
| Mean | 6.29 | 9.27 |  |  |  |
| Median (Q1-Q3) | 6.11 (5.38-7.51) | 7.77 (7.11-8.52) |  |  |  |
| Min - Max | 1.73 - 9.47 | 5.86 - 22.82 |  |  |  |
| **hsa-miR-224-5p** |  |  | 1.76 | 0.628 | 0.812 |
| N | 10 | 9 |  |  |  |
| N missing | 0 | 0 |  |  |  |
| Mean | 0.015 | 0.008 |  |  |  |
| Median (Q1-Q3) | 0.008 (0.005-0.019) | 0.005 (0.000-0.013) |  |  |  |
| Min - Max | 0.000 - 0.058 | 0.000 - 0.023 |  |  |  |
| **hsa-miR-23a-3p** |  |  | 0.863 | 0.095 | 0.474 |
| N | 10 | 9 |  |  |  |
| N missing | 0 | 0 |  |  |  |
| Mean | 2.02 | 2.34 |  |  |  |
| Median (Q1-Q3) | 2.03 (1.68-2.30) | 2.22 (2.18-2.32) |  |  |  |
| Min - Max | 1.46 - 2.65 | 1.93 - 3.07 |  |  |  |
| **hsa-miR-25-3p** |  |  | 1.42 | 0.502 | 0.747 |
| N | 10 | 9 |  |  |  |
| N missing | 0 | 0 |  |  |  |
| Mean | 3.69 | 2.57 |  |  |  |
| Median (Q1-Q3) | 2.51 (1.75-4.77) | 2.13 (1.41-2.59) |  |  |  |
| Min - Max | 1.47 - 8.22 | 0.56 - 7.45 |  |  |  |
| **hsa-miR-27a-3p** |  |  | 0.779 | 0.039 | 0.474 |
| N | 10 | 9 |  |  |  |
| N missing | 0 | 0 |  |  |  |
| Mean | 0.86 | 1.09 |  |  |  |
| Median (Q1-Q3) | 0.87 (0.79-0.96) | 0.96 (0.92-1.18) |  |  |  |
| Min - Max | 0.58 - 0.98 | 0.80 - 1.84 |  |  |  |
| **hsa-miR-296-5p** |  |  | 1.85 | 0.020 | 0.462 |
| N | 10 | 9 |  |  |  |
| N missing | 0 | 0 |  |  |  |
| Mean | 0.011 | 0.006 |  |  |  |
| Median (Q1-Q3) | 0.011 (0.008-0.012) | 0.006 (0.005-0.007) |  |  |  |
| Min - Max | 0.004 - 0.025 | 0.004 - 0.010 |  |  |  |
| **hsa-miR-29a-3p** |  |  | 1.06 | 0.796 | 0.912 |
| N | 10 | 9 |  |  |  |
| N missing | 0 | 0 |  |  |  |
| Mean | 0.30 | 0.29 |  |  |  |
| Median (Q1-Q3) | 0.29 (0.20-0.44) | 0.27 (0.21-0.37) |  |  |  |
| Min - Max | 0.11 - 0.48 | 0.13 - 0.48 |  |  |  |
| **hsa-miR-30d-5p** |  |  | 1.10 | 0.571 | 0.795 |
| N | 10 | 9 |  |  |  |
| N missing | 0 | 0 |  |  |  |
| Mean | 0.51 | 0.47 |  |  |  |
| Median (Q1-Q3) | 0.49 (0.46-0.57) | 0.45 (0.41-0.53) |  |  |  |
| Min - Max | 0.28 - 0.87 | 0.33 - 0.63 |  |  |  |
| **hsa-miR-34a-5p** |  |  | 0.807 | 0.272 | 0.568 |
| N | 10 | 9 |  |  |  |
| N missing | 0 | 0 |  |  |  |
| Mean | 0.004 | 0.006 |  |  |  |
| Median (Q1-Q3) | 0.004 (0.000-0.006) | 0.004 (0.003-0.007) |  |  |  |
| Min - Max | 0.000 - 0.018 | 0.000 - 0.017 |  |  |  |
| **hsa-miR-375-3p** |  |  | 0.802 | 0.455 | 0.747 |
| N | 10 | 9 |  |  |  |
| N missing | 0 | 0 |  |  |  |
| Mean | 0.012 | 0.015 |  |  |  |
| Median (Q1-Q3) | 0.010 (0.005-0.013) | 0.010 (0.007-0.022) |  |  |  |
| Min - Max | 0.000 - 0.035 | 0.002 - 0.035 |  |  |  |
| **hsa-miR-423-5p** |  |  | 1.92 | 0.985 | 0.991 |
| N | 10 | 9 |  |  |  |
| N missing | 0 | 0 |  |  |  |
| Mean | 0.72 | 0.41 |  |  |  |
| Median (Q1-Q3) | 0.34 (0.23-0.63) | 0.41 (0.21-0.44) |  |  |  |
| Min - Max | 0.19 - 3.71 | 0.13 - 0.95 |  |  |  |
| **hsa-miR-574-3p** |  |  | 1.07 | 0.864 | 0.929 |
| N | 10 | 9 |  |  |  |
| N missing | 0 | 0 |  |  |  |
| Mean | 0.013 | 0.012 |  |  |  |
| Median (Q1-Q3) | 0.012 (0.009-0.016) | 0.012 (0.011-0.014) |  |  |  |
| Min - Max | 0.006 - 0.025 | 0.000 - 0.028 |  |  |  |
| **hsa-miR-885-5p** |  |  | 1.28 | 0.835 | 0.912 |
| N | 10 | 9 |  |  |  |
| N missing | 0 | 0 |  |  |  |
| Mean | 0.020 | 0.017 |  |  |  |
| Median (Q1-Q3) | 0.012 (0.005-0.027) | 0.012 (0.005-0.026) |  |  |  |
| Min - Max | 0.000 - 0.084 | 0.003 - 0.040 |  |  |  |
| **hsa-miR-92a-3p** |  |  | 1.81 | 0.326 | 0.626 |
| N | 10 | 9 |  |  |  |
| N missing | 0 | 0 |  |  |  |
| Mean | 14 | 8 |  |  |  |
| Median (Q1-Q3) | 9 (6-17) | 8 (4-10) |  |  |  |
| Min - Max | 5 - 48 | 2 - 18 |  |  |  |
| **hsa-miR-93-5p** |  |  | 1.48 | 0.139 | 0.474 |
| N | 10 | 9 |  |  |  |
| N missing | 0 | 0 |  |  |  |
| Mean | 1.88 | 1.27 |  |  |  |
| Median (Q1-Q3) | 1.69 (1.27-2.20) | 1.13 (0.93-1.40) |  |  |  |
| Min - Max | 0.54 - 3.68 | 0.47 - 2.23 |  |  |  |
| **hsa-let-7c-5p** |  |  | 0.910 | 0.490 | 0.747 |
| N | 10 | 9 |  |  |  |
| N missing | 0 | 0 |  |  |  |
| Mean | 0.38 | 0.42 |  |  |  |
| Median (Q1-Q3) | 0.38 (0.33-0.43) | 0.39 (0.33-0.45) |  |  |  |
| Min - Max | 0.20 - 0.51 | 0.30 - 0.66 |  |  |  |
| **hsa-miR-107** |  |  | 1.31 | 0.133 | 0.474 |
| N | 10 | 9 |  |  |  |
| N missing | 0 | 0 |  |  |  |
| Mean | 1.27 | 0.98 |  |  |  |
| Median (Q1-Q3) | 1.13 (0.95-1.25) | 0.91 (0.89-1.13) |  |  |  |
| Min - Max | 0.88 - 2.83 | 0.72 - 1.23 |  |  |  |
| **hsa-miR-10a-5p** |  |  | 0.631 | 0.134 | 0.474 |
| N | 10 | 9 |  |  |  |
| N missing | 0 | 0 |  |  |  |
| Mean | 0.0033 | 0.0052 |  |  |  |
| Median (Q1-Q3) | 0.0034 (0.0014-0.0041) | 0.0040 (0.0029-0.0060) |  |  |  |
| Min - Max | 0.0010 - 0.0076 | 0.0024 - 0.0131 |  |  |  |
| **hsa-miR-128-3p** |  |  | 1.61 | 0.342 | 0.639 |
| N | 10 | 9 |  |  |  |
| N missing | 0 | 0 |  |  |  |
| Mean | 0.025 | 0.015 |  |  |  |
| Median (Q1-Q3) | 0.016 (0.012-0.033) | 0.011 (0.010-0.018) |  |  |  |
| Min - Max | 0.010 - 0.063 | 0.005 - 0.040 |  |  |  |
| **hsa-miR-130b-3p** |  |  | 2.92 | 0.322 | 0.626 |
| N | 10 | 9 |  |  |  |
| N missing | 0 | 0 |  |  |  |
| Mean | 0.13 | 0.05 |  |  |  |
| Median (Q1-Q3) | 0.06 (0.04-0.11) | 0.03 (0.03-0.04) |  |  |  |
| Min - Max | 0.02 - 0.68 | 0.02 - 0.12 |  |  |  |
| **hsa-miR-145-5p** |  |  | 0.686 | 0.100 | 0.474 |
| N | 10 | 9 |  |  |  |
| N missing | 0 | 0 |  |  |  |
| Mean | 0.18 | 0.27 |  |  |  |
| Median (Q1-Q3) | 0.15 (0.11-0.19) | 0.27 (0.11-0.36) |  |  |  |
| Min - Max | 0.08 - 0.52 | 0.07 - 0.56 |  |  |  |
| **hsa-miR-148a-3p** |  |  | 1.40 | 0.636 | 0.812 |
| N | 10 | 9 |  |  |  |
| N missing | 0 | 0 |  |  |  |
| Mean | 0.53 | 0.39 |  |  |  |
| Median (Q1-Q3) | 0.43 (0.31-0.55) | 0.36 (0.25-0.55) |  |  |  |
| Min - Max | 0.20 - 1.59 | 0.15 - 0.71 |  |  |  |
| **hsa-miR-15a-5p** |  |  | 1.84 | 0.816 | 0.912 |
| N | 10 | 9 |  |  |  |
| N missing | 0 | 0 |  |  |  |
| Mean | 6.65 | 3.82 |  |  |  |
| Median (Q1-Q3) | 3.11 (2.30-7.24) | 3.48 (1.77-4.41) |  |  |  |
| Min - Max | 2.05 - 29.85 | 0.87 - 8.32 |  |  |  |
| **hsa-miR-193a-5p** |  |  | 1.23 | 0.621 | 0.812 |
| N | 9 | 9 |  |  |  |
| N missing | 1 | 0 |  |  |  |
| Mean | 0.026 | 0.021 |  |  |  |
| Median (Q1-Q3) | 0.018 (0.010-0.038) | 0.015 (0.010-0.034) |  |  |  |
| Min - Max | 0.000 - 0.072 | 0.003 - 0.059 |  |  |  |
| **hsa-miR-204-5p** |  |  | 0.421 | 0.122 | 0.474 |
| N | 10 | 8 |  |  |  |
| N missing | 0 | 1 |  |  |  |
| Mean | 0.0020 | 0.0047 |  |  |  |
| Median (Q1-Q3) | 0.0013 (0.0000-0.0034) | 0.0045 (0.0012-0.0079) |  |  |  |
| Min - Max | 0.0000 - 0.0053 | 0.0000 - 0.0103 |  |  |  |
| **hsa-miR-26b-5p** |  |  | 1.22 | 0.219 | 0.554 |
| N | 10 | 9 |  |  |  |
| N missing | 0 | 0 |  |  |  |
| Mean | 0.81 | 0.67 |  |  |  |
| Median (Q1-Q3) | 0.82 (0.68-1.03) | 0.62 (0.56-0.71) |  |  |  |
| Min - Max | 0.33 - 1.22 | 0.42 - 1.03 |  |  |  |
| **hsa-miR-30e-5p** |  |  | 1.20 | 0.470 | 0.747 |
| N | 10 | 9 |  |  |  |
| N missing | 0 | 0 |  |  |  |
| Mean | 0.19 | 0.15 |  |  |  |
| Median (Q1-Q3) | 0.16 (0.15-0.24) | 0.12 (0.10-0.17) |  |  |  |
| Min - Max | 0.07 - 0.34 | 0.09 - 0.34 |  |  |  |
| **hsa-miR-374a-5p** |  |  | 1.06 | 0.835 | 0.912 |
| N | 10 | 9 |  |  |  |
| N missing | 0 | 0 |  |  |  |
| Mean | 0.34 | 0.32 |  |  |  |
| Median (Q1-Q3) | 0.40 (0.20-0.42) | 0.30 (0.23-0.35) |  |  |  |
| Min - Max | 0.15 - 0.57 | 0.11 - 0.71 |  |  |  |
| **hsa-miR-376c-3p** |  |  | 0.520 | 0.008 | 0.284 |
| N | 10 | 9 |  |  |  |
| N missing | 0 | 0 |  |  |  |
| Mean | 0.05 | 0.11 |  |  |  |
| Median (Q1-Q3) | 0.05 (0.04-0.07) | 0.10 (0.08-0.14) |  |  |  |
| Min - Max | 0.02 - 0.10 | 0.05 - 0.15 |  |  |  |
| **hsa-miR-7-5p** |  |  | 1.96 | 0.081 | 0.474 |
| N | 10 | 9 |  |  |  |
| N missing | 0 | 0 |  |  |  |
| Mean | 0.027 | 0.014 |  |  |  |
| Median (Q1-Q3) | 0.024 (0.014-0.034) | 0.010 (0.006-0.024) |  |  |  |
| Min - Max | 0.008 - 0.062 | 0.002 - 0.029 |  |  |  |
| **hsa-miR-96-5p** |  |  | 0.779 | 0.368 | 0.670 |
| N | 10 | 7 |  |  |  |
| N missing | 0 | 2 |  |  |  |
| Mean | 0.011 | 0.014 |  |  |  |
| Median (Q1-Q3) | 0.009 (0.005-0.016) | 0.013 (0.009-0.017) |  |  |  |
| Min - Max | 0.000 - 0.028 | 0.003 - 0.030 |  |  |  |
| **hsa-miR-103a-3p** |  |  | 1.24 | 0.122 | 0.474 |
| N | 10 | 9 |  |  |  |
| N missing | 0 | 0 |  |  |  |
| Mean | 1.56 | 1.26 |  |  |  |
| Median (Q1-Q3) | 1.42 (1.32-1.61) | 1.25 (0.97-1.52) |  |  |  |
| Min - Max | 1.02 - 2.93 | 0.89 - 1.63 |  |  |  |
| **hsa-miR-15b-5p** |  |  | 1.22 | 0.505 | 0.747 |
| N | 10 | 9 |  |  |  |
| N missing | 0 | 0 |  |  |  |
| Mean | 1.67 | 1.37 |  |  |  |
| Median (Q1-Q3) | 1.64 (1.10-1.72) | 1.11 (1.06-1.44) |  |  |  |
| Min - Max | 1.05 - 3.01 | 0.84 - 2.56 |  |  |  |
| **hsa-miR-16-5p** |  |  | 1.52 | 0.272 | 0.568 |
| N | 10 | 9 |  |  |  |
| N missing | 0 | 0 |  |  |  |
| Mean | 39 | 25 |  |  |  |
| Median (Q1-Q3) | 31 (22-50) | 20 (13-25) |  |  |  |
| Min - Max | 17 - 79 | 7 - 69 |  |  |  |
| **hsa-miR-191-5p** |  |  | 1.10 | 0.490 | 0.747 |
| N | 10 | 9 |  |  |  |
| N missing | 0 | 0 |  |  |  |
| Mean | 1.09 | 0.99 |  |  |  |
| Median (Q1-Q3) | 1.06 (0.91-1.15) | 1.06 (0.90-1.15) |  |  |  |
| Min - Max | 0.65 - 1.81 | 0.72 - 1.17 |  |  |  |
| **hsa-miR-22-3p** |  |  | 1.52 | 0.200 | 0.542 |
| N | 10 | 9 |  |  |  |
| N missing | 0 | 0 |  |  |  |
| Mean | 1.80 | 1.21 |  |  |  |
| Median (Q1-Q3) | 1.49 (1.16-1.90) | 1.17 (0.96-1.44) |  |  |  |
| Min - Max | 0.61 - 5.24 | 0.58 - 2.12 |  |  |  |
| **hsa-miR-24-3p** |  |  | 0.877 | 0.147 | 0.474 |
| N | 10 | 9 |  |  |  |
| N missing | 0 | 0 |  |  |  |
| Mean | 0.89 | 1.02 |  |  |  |
| Median (Q1-Q3) | 0.93 (0.79-0.98) | 0.99 (0.88-1.07) |  |  |  |
| Min - Max | 0.64 - 1.07 | 0.86 - 1.38 |  |  |  |
| **hsa-miR-26a-5p** |  |  | 0.982 | 0.927 | 0.968 |
| N | 10 | 9 |  |  |  |
| N missing | 0 | 0 |  |  |  |
| Mean | 0.79 | 0.80 |  |  |  |
| Median (Q1-Q3) | 0.79 (0.64-0.94) | 0.86 (0.61-0.95) |  |  |  |
| Min - Max | 0.28 - 1.23 | 0.60 - 1.05 |  |  |  |
| ^1^Permutation Yuen-Welch t test | | | | | |
| ^2^Benjamini & Hochberg correction for multiple testing | | | | | |
