# Supplementary material for: Circulating miRNA profiles and the risk of hemorrhagic transformation after thrombolytic treatment of acute ischemic stroke: a pilot study
Source: Front Neurol. 2024 Jun 12;15:1399345. doi: 10.3389/fneur.2024.1399345 (PMC11210454; doi:10.3389/fneur.2024.1399345)

***Supplementary Table 4 Expression of selected miRNAs in various organs according to the TissueAtlas.***


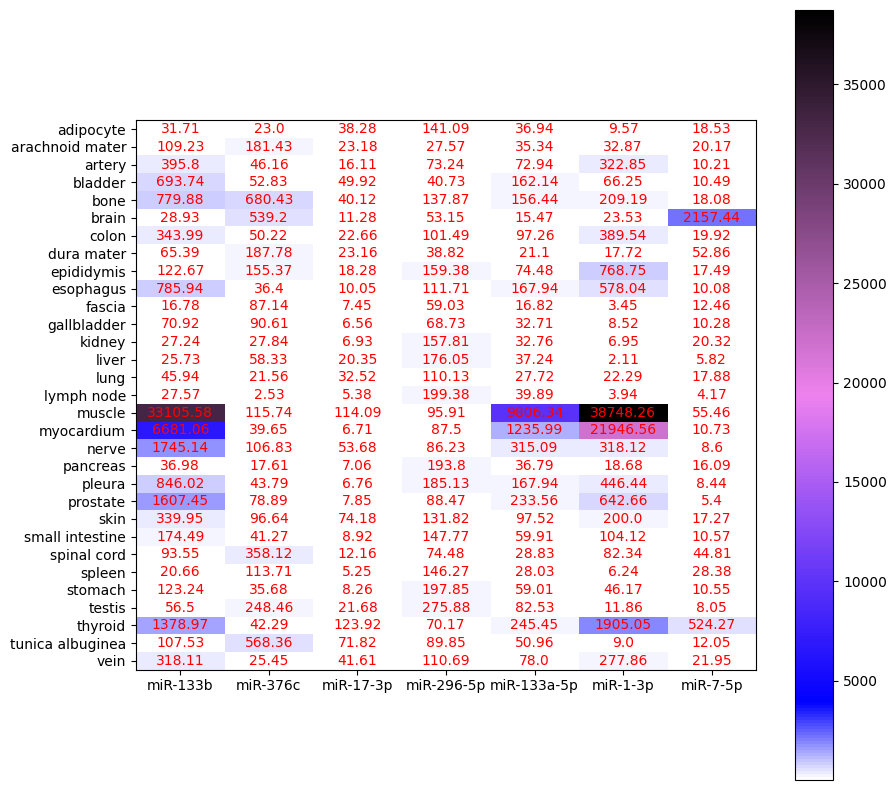

Supplement: SUPPLEMENTARY TABLE S4 — Expression of selected miRNAs in various organs according to the TissueAtlas. [file Table_4.docx]
